# Autism Spectrum Disorder and a De Novo Kcnq2 Gene Mutation: A Case Report

**DOI:** 10.3390/pediatric14020027

**Published:** 2022-04-24

**Authors:** Martina Siracusano, Claudia Marcovecchio, Assia Riccioni, Caterina Dante, Luigi Mazzone

**Affiliations:** 1 Department of Biomedicine and Prevention, University of Rome Tor Vergata, 00133 Rome, Italy; 2 Child Neurology and Psychiatry Unit, Department of Neurosciences, Policlinico Tor Vergata Foundation Hospital, 00133 Rome, Italy; claudia.marcovecchio3@gmail.com (C.M.); assiariccioni@gmail.com (A.R.); catedc@gmail.com (C.D.); luigi.mazzone@uniroma2.it (L.M.); 3 Systems Medicine Department, University of Rome Tor Vergata, 00133 Rome, Italy

**Keywords:** autism, neuropsychological phenotype, development, social behavior, KCNQ2, epilepsy, neuronal excitability

## Abstract

The KCNQ2 gene, encoding for the K_v_7.2 subunits of the K_v_7 voltage potassium channel, is involved in the modulation of neuronal excitability and plays a crucial role in brain morphogenesis and maturation during embryonic life. De novo heterozygous mutations in KCNQ2 genes are associated with early-onset epileptic encephalopathy and neurodevelopmental disorders including developmental delay and intellectual disability. However, little is known about the socio-communicative phenotype of children affected by the KCNQ2 mutation, and a detailed behavioral characterization focused on autistic symptoms has not yet been conducted. This case report describes the clinical behavioral phenotype of a 6-year-old boy carrying a de novo heterozygous KCNQ2 mutation, affected by early-onset seizures and autism spectrum disorder (ASD). We performed a neuropsychiatric assessment of cognitive, adaptive, socio-communicative and autistic symptoms through the administration of standardized tools. The main contribution of this case report is to provide a detailed developmental and behavioral characterization focused on ASD symptoms in a child with [c.812 G > A; p. (Gly271Asp)]mutation in the KCNQ2 gene.

## 1. Introduction

The KCNQ2 gene (20p13.3) plays a crucial role in the modulation of neuronal excitability, and in regulating neuronal proliferation, differentiation and brain maturation since prenatal life [1,2,3]. Specifically, KCNQ2 encodes for the K_v_7.2 subunits of the k_v_7 voltage potassium channel, which regulates the resting membrane potential (RMP) [1]. KCNQ/K_v_7 channels are highly expressed in many regions of the brain implicated in higher cognitive and executive functions such as the hippocampus, prefrontal cortex, reticular thalamic nucleus and striatal neurons, where they modulate presynaptic and/or postsynaptic activity [4]. Severe dysfunction of KCNQ/K_v_7 channels determines neuronal hyper-excitability leading to elevated seizure susceptibility; furthermore, due to the crucial role of K_v_7 channels on neurodevelopment during embryonic life, it is not surprising to register the association between KCNQ/K_v_7 disruption and developmental delay (i.e., delayed motor and language milestones, intellectual disability) [1]. Indeed, heterozygous mutations in KCNQ2 genes have been related to early-onset epilepsy and neurodevelopmental disorders [1]. Specifically, inherited mutations cause benign familial neonatal encephalopathy (BFNE), characterized by neonatal seizures spontaneously remitting after several weeks/months, without psychomotor and intellectual impairment; whilst de novo mutations are associated with early-onset epileptic encephalopathy (EE) and neurodevelopmental disorders including developmental delay and intellectual disability [4,5]. 

In the last few years, the KCNQ2 gene has gained interest in the field of autism spectrum disorder (ASD), an early onset and lifelong neurodevelopmental disorder characterized by impairment in social communication, restricted interests, and repetitive behaviors [6].

Both animal and human models have in fact investigated the social, developmental and behavioral correlates of loss-of-function KCNQ2 mutations with promising results [4,7,8]. In a recent study, Kim et al. found that, in comparison to wild-type rodents, KCNQ2^+/−^ mice (heterozygous null for the gene) were characterized by deficits in social recognition and motivation, increased aggressiveness, enhanced repetitive and compulsive-like behavior together with elevated seizure susceptibility [4].

It is worth noting that human studies investigating the genotype-phenotype association in children with KCNQ2 encephalopathy have reported interesting findings [7,8]. In particular, Milh et al. and Millichap et al. found that, within their samples, in addition to the cognitive and developmental disability, some children displayed autistic features (i.e., repetitive movements, poor eye contact, language and social impairment) [7,8]. However, the authors did not perform a specific evaluation through standardized tools aimed at investigating autistic symptoms.

To our knowledge, detailed developmental and behavioral characterization—focused on autistic symptoms—of children with heterozygous loss of KCNQ2, has not been reported in the literature.

Therefore, we report the case of a 6-year-old child with a de novo heterozygous mutation in the KCNQ2 gene and affected by early-onset epilepsy and ASD.

## 2. Case Presentation

This clinical case describes a 6-year-old Italian male child, carrying a [c.812 G > A; p. (Gly271Asp)] KCNQ2 mutation with developmental delay, who referred to our Child Psychiatry Unit for a neuropsychiatric assessment of cognitive skills and social behavior. 

He was an only child of non-consanguineous white parents, with a negative medical history for neurological and psychiatric diseases, born at 29 weeks gestation by emergency caesarian section for preterm premature rupture of membrane (PPROM) and oligoidramnios. His birth weight was 1290 g (50–10th percentile), body length was 41 cm (50–90th percentile), head circumference was 28 cm (50–90th percentile), and APGAR score was five at the first minute and seven at the fifth minute. After birth, the newborn suffered from cardiorespiratory depression, cyanosis and hypotonia, for which he was intubated for 12 h. Hyperglycemia and neonatal jaundice were also referred. The neonatal period was characterized, at the 15th day of life, by clusters of generalized tonic seizures associated with desaturation. Intravenous treatment with Phenytoin and Pyridoxine was administered with mild clinical improvement. Maintenance therapy was subsequently administered with phenytoin and levetiracetam until the age of 13 months, with gradual disappearance of seizures.

Due to the early onset of seizures, he underwent genetic counseling and next generation sequencing (NGS), which displayed the de novo heterozygous KCNQ2 mutation [c.812 G > A; p. (Gly271Asp)]. Moreover, a brain MRI was performed at birth and repeated at 2 months, not showing pathological findings.

During infancy, the child underwent an EEG at 3 months which showed diffuse epileptic activity on the anterior regions with right prevalence. The child repeated the EEG at 7 months without revealing epileptiform abnormalities. Follow-up medical examination and instrumental investigation (EEG, eye examination) were performed with annual frequency. The last EEG and brain MRI (performed at 5 years of age) did not report significant alterations. 

At the age of 4 years, the child underwent the following genetic examinations: a chromosomal microarray analysis (CMA), not revealing microdeletions and microduplications; telomeres analysis (multiplex ligation-dependent probe amplification MPLA-P070-B2); metilation test of 15q11-13 region using MLPA-ME028-B2, revealing normal condition; analysis of the cariotype (46 XY).

A history of mild motor developmental delay was reported: autonomous walking occurred at 18 months. First word was spoken at 12 months, followed by verbal development interruption and regression. Sleep/wake cycle was characterized by difficulty in falling asleep, nocturnal awakenings and cosleeping with parents, together with benefits after melatonin supplementation (1 mg). Feeding was regular but constipation was referred.

First parental concerns regarding child behaviors and social development were reported at 18 months of age for the onset of atypical behaviors: deficits in social interactions and communication skills, repetitive interests, stereotyped hand movements and hyperactivity. Therefore, at the age of 2 years and 7 months, the child underwent a first neuropsychiatric assessment showing a developmental delay with an age equivalent of 20 months on the Griffiths Scale 3rd edition, ref. [9]—a standardized tool that provides an overall measure of a child’s development—with the subsequent prescription of child physiotherapy and speech therapy without a significant improvement. 

During preschool age, speech delay persisted, and parents reported deficits in reciprocal social communication, social interaction, restrictive patterns of behavior and interests, as well as worsening behavioral problems (irritability, frustration, aggressiveness and disruptive behaviors).

## 3. Neuropsychiatric Assessment

For the reasons reported above, at 6 years of age the child underwent an in-depth clinical evaluation in our Child Psychiatry Unit, including an assessment of cognitive, adaptive, socio-communicative and behavioral skills, performed through the administration of the following standardized tools (see Table A1). On our clinical examination, the child was not taking medication (he was seizure-free since 24 months of age; the last EEG was at 5 years, without significant pathological findings).

To start with, cognitive skills were measured using the non-verbal Leiter International Performance Scale, Third Edition (Leiter-3) [10], revealing a non-verbal intellectual quotient (IQ) of 79 (borderline score). Adaptive skills were assessed through the parental questionnaire, Adaptive Behavior Assessment System, Second Edition (ABAS-II) [11], reporting a functioning below the average in all domains (General Adaptive, Conceptual, Social, Practical) (Table A1).

ASD symptoms were evaluated according to the DSM-5 criteria and through the administration of the Autism Diagnostic Observation Schedule, Second Edition (ADOS-2) [12], performed by a licensed clinician. The child underwent the Module 2 of the ADOS-2, suitable for children with a verbal language composed of phrases. The diagnostic algorithm is organized in two main areas: social affect (SA) and restricted and repetitive behavior (RRB). The total score obtained (SA 10 + RBB 2 = 12) exceeded the cut-off (9) for the diagnosis of autism and, according to the ADOS-2 Calibrated Severity Score (ADOS-CSS), was suggestive of a moderate level of ASD symptoms severity.

Finally, parents filled in the questionnaires described below in order to evaluate the child’s social relationships and problematic behaviors. 

The Social Responsiveness Scale (SRS) [13], a 65-item rating scale which measures child’s social impairment assessing social awareness, social information processing, reciprocal social communication and autistic traits, displayed a severe deficit of the child social relationship with a significant impairment of everyday functioning (see Table A1).

The Child Behavior Check-list (CBCL 6–18), a behavior assessment measure [14], showed significant problematic behaviors in the following domains: internalizing problems, affective problems and total problems; whereas borderline scores emerged in the anxiety problems, externalizing problems and withdrawn/depression subscales (see Table A1).

## 4. Discussion

The association between the KCNQ2 mutation, epilectic encephalopaty and developmental delay [1,4,7,8] is nowadays overt. However, little is known about developmental and behavioral characterization of the ASD clinical phenotype associated with this mutation. 

In this report we describe the case of a 6-year-old child with a [c.812 G > A; p. (Gly271Asp)]mutation of the KCNQ2 gene, affected by early-onset seizures, developmental delay and ASD.

Our results are concordant with the emerging findings of a clinical autistic phenotype within individuals carrying the KCNQ2 gene mutation [7,8]. In particular, Milh et al, conducted a study on 71 patients with early onset epileptic encephalopaties (EOEEs) aiming to describe the role of the KCNQ2 gene in the diagnosis workup of EOEEs, and to characterize clinical and EEG features associated with the mutation. Screening this cohort, they found that 16 individuals had a de novo mutation in the KCNQ2 gene and 30% of them showed autistic features. However, the authors did not provide a clear definition of “autistic features” and they did not employ standardized instruments, as we did (specifically a parental questionnaire or objective clinical evaluation performed by specialists) in order to investigate and quantify these symptoms [7].

Furthermore, Millichap et al. retrospectively evaluated the genotype-phenotype relationships of 23 patients with KCNQ2 encephalopathy, followed by a team of pediatric neurologists and geneticists who specifically investigated the developmental impairment (defined as mild, moderate and severe on the basis of milestones achievement at follow-up). In particular, normal development was not described in any of the patients included in the study, whilst nine individuals were characterized by mild-moderate developmental impairment and the majority was severely impaired. Interestingly, the authors described unspecified autistic features in some of the patients characterized by a mild-moderate developmental impairment, although without performing a standardized evaluation of such symptoms [8].

Even if our study included only one child, who was prematurely born, the contribution that this case report may give to the actual literature with a subsequent impact on both clinical and therapeutical issues is worth noting. We are aware that the condition of prematurity of the child could have influenced his neurodevelopmental outcome, but our case report does not want to claim an entire association of ASD with the KCNQ2 gene mutation. The present manuscript aims to draw attention to the need to provide an early evaluation of behavioral features for children carrying this mutation with a specific focus on autism symptoms, using standardized measures.

In comparison to the research described above, we provided a detailed characterization of the ASD clinical phenotype using standardized tools—the gold standard for the assessment of autistic symptoms—including the Autism Diagnostic Observation Schedule (ADOS-2), the Social Communication Questionnaire (SCQ) and the Social Responsiveness Scale (SRS). As a matter of fact, the early identification of ASD symptoms within individuals affected by de novo heterozygous mutation in the KCNQ2 gene could lead to early intervention with a possible improvement of developmental and behavioral outcomes in this population.

Therefore, from a clinical point of view, our results recommend to perform a screening of ASD symptoms on children with early-onset epilepsy who present [c.812 G > A; p. (Gly271Asp)] KCNQ2 gene mutation.

Furthermore, we may speculate that our findings, in accordance with previous clinical studies [7,8] and animal models [4], suggest that a dysfunction of KCNQ/K_v_7 channels (whose subunits are coded by the KCNQ2 gene), determining neuronal hyper-excitability, may contribute to ASD behaviors [4]. This is concordant with the known association between ASD and excitatory/inhibitory (E/I) imbalances [15]. Specifically, several studies have suggested that an overall increased ratio of E/I could lead not only to increased epileptic susceptibility but could also have a role in the development of atypical behaviors [15]. In this context, the modulation of E/I may represent a promising therapeutic target in animal models of ASD [16].

## 5. Conclusions

To the best of our knowledge, this is the first clinical description of ASD symptoms performed through standardized instruments in a child carrying a de novo heterozygous mutation in the KCNQ2 gene.

Further studies on wider samples, including children not prematurely born, are needed in order to provide an effective association between ASD and KCNQ2 gene mutations.

## Data Availability

Data sharing is not applicable to this article as no datasets were generated or analyzed during the current study.

## Appendix A

**Table A1:** Neuropsychiatric Assessment.

SKILLS	TOOLS	RESULTS
**Cognitive**	LEITER-3	IQ 79
	ABAS-II	**Composite score:** General Adaptive Domain 52; Conceptual Domain 61; Social Domain 62; Practical Domain 46
**Socio-communicative behavior**	ADOS-2 (Module 2)	SA 10; RRB 2; Total 12; CSS 6
	SRS	**T score:** Total 81; Social Awareness 69; Social Cognition 72; Social Communication 82; Social Motivation 79; Autistic Manerism 72
	CBCL (6-18 years)	**T score:** Affective Problems = 70; Anxiety = 65; Tought = 70; Withdrawn/Depression = 66; Internalizing = 65; Externalizing = 62

Main findings of the neuropsychiatric assessment (cognitive skills, including IQ and adaptive functioning; socio-communicative behavior) performed in our Child Psychiatric Unit at the age of 6 years. ABAS-II, Adaptive Behavior Assessment System, Second Edition; ADOS-2, Autism Diagnostic Observation Schedule, Second Edition; CBCL, Child Behavior Checklist; CSS, Calibrated Severity Score; IQ, intellectual quotient; LEITER-3, Leiter International Performance Scale, Third Edition; RBB, restricted and repetitive behavior; SA, social affect; SRS Social Responsiveness Scale.

## Appendix B

List of abbreviations:

- ABAS-II = Adaptive Behavior Assessment System-Second Edition;
- ADOS-2 = Autism Diagnostic Observation Schedule-Second Edition;
- ASD = Autism Spectrum Disorder;
- BFNE = Benign Familial Neonatal Encephalopathy;
- CBCL = Child Behavior Checklist;
- CSS = Calibrated Severity Score;
- EE= Epileptic Encephalopathy;
- EEG = Electroencephalography;
- E/I = excitatory/inhibitory imbalance
- EOEEs = Early Onset Epileptic Encephalopathies;
- IQ = Intellectual Quotient;
- MRI = Magnetic Resonance Imaging;
- NGS = Next Generation Sequencing;
- PPROM = Preterm Premature Rupture of Membrane;
- RRB = Restricted and Repetitive Behavior;
- SA = Social Affect;
- SRS = Social Responsiveness Scale.
